# Serum S100B Is Related to Illness Duration and Clinical Symptoms in Schizophrenia—A Meta-Regression Analysis

**DOI:** 10.3389/fncel.2016.00046

**Published:** 2016-02-25

**Authors:** Katharina Schümberg, Maryna Polyakova, Johann Steiner, Matthias L. Schroeter

**Affiliations:** ^1^Department of Cognitive Neurology, Max Planck Institute for Human Cognitive and Brain SciencesLeipzig, Germany; ^2^Department of Psychiatry, University of MagdeburgMagdeburg, Germany; ^3^Clinic for Cognitive Neurology, University of LeipzigLeipzig, Germany; ^4^LIFE—Leipzig Research Center for Civilization Diseases, University of LeipzigLeipzig, Germany; ^5^German Consortium for Frontotemporal Lobar DegenerationUlm, Germany

**Keywords:** glia, meta-analysis, S100B, schizophrenia, serum marker

## Abstract

S100B has been linked to glial pathology in several psychiatric disorders. Previous studies found higher S100B serum levels in patients with schizophrenia compared to healthy controls, and a number of covariates influencing the size of this effect have been proposed in the literature. Here, we conducted a meta-analysis and meta-regression analysis on alterations of serum S100B in schizophrenia in comparison with healthy control subjects. The meta-analysis followed the Preferred Reporting Items for Systematic Reviews and Meta-Analyses (PRISMA) statement to guarantee a high quality and reproducibility. With strict inclusion criteria 19 original studies could be included in the quantitative meta-analysis, comprising a total of 766 patients and 607 healthy control subjects. The meta-analysis confirmed higher values of the glial serum marker S100B in schizophrenia if compared with control subjects. Meta-regression analyses revealed significant effects of illness duration and clinical symptomatology, in particular the total score of the Positive and Negative Syndrome Scale (PANSS), on serum S100B levels in schizophrenia. In sum, results confirm glial pathology in schizophrenia that is modulated by illness duration and related to clinical symptomatology. Further studies are needed to investigate mechanisms and mediating factors related to these findings.

## Introduction

S100B is a calcium-binding protein which can be secreted by astroglia and oligodendroglia (e.g., Donato, 2001; Steiner et al., 2007, 2008; Donato et al., 2009). In the brain, it is generally assumed to be neurotrophic in nanomolar concentrations due to its activation of neural growth factor pathways and up-regulation of anti-apoptotic factors. In micromolar concentrations however, it becomes neurotoxic, interacting with pro-inflammatory cytokines like interleukin (IL)-1, IL-6, tumor necrosis factor (TNF) alpha, up-regulating the expression of pro-apoptotic factors as well as inducing nitric oxide synthase and cyclooxygenase 2 expression (Donato, 2001; Van Eldik and Wainwright, 2003; Donato et al., 2009; Bianchi et al., 2010). Thus, S100B has been linked to the presence of inflammatory processes in the brain, either after injury or due to primary inflammation (Sen and Belli, 2007). Furthermore, certain neuropsychiatric disorders, among them schizophrenia, have been found to be associated with elevated serum and cerebrospinal fluid (CSF) levels of S100B (e.g., Rothermundt et al., 2004a), thus implying immunoreactive glial processes as important in either their genesis or progression. Although serum S100B might be associated with other conditions such as blood-brain barrier disruption, weight changes or neurological diseases, S100B changes in schizophrenia are lower than would be expected in neurological diseases with brain injury (Schroeter et al., 2009; Steiner et al., 2010b).

Here, we conducted a systematic and quantitative meta-analysis of changes in serum S100B in schizophrenia, which extends former meta-analyses on this issue (Schroeter et al., 2003, 2009; Schroeter and Steiner, 2009; Aleksovska et al., 2014) by including further studies and accordingly, increasing statistical power and evidence. Based on previous studies, we hypothesized higher S100B in schizophrenia when compared to healthy controls. Additionally, we investigated effects of medication and applied meta-regression analyses to investigate effects of clinical parameters on serum S100B. We hypothesized here an association of S100B with the duration of illness as recent studies have shown progression of astrocytes’ dystrophy/swelling and of oligodendrocyte-related disturbances of cerebral connectivity with duration of illness (Kolomeets and Uranova, 2010; Bernstein et al., 2015; Yao et al., 2015). Moreover, we hypothesized an association of S100B with negative symptoms as shown in previous studies (Rothermundt et al., 2001, 2004b; Schroeter et al., 2003; Schmitt et al., 2005).

## Materials and Methods

### General Study Selection Criteria

The meta-analysis was conducted according to the Preferred Reporting Items for Systematic Reviews and Meta-Analyses (PRISMA) statement to guarantee a high quality and reproducibility of the meta-analysis (Moher et al., 2009). The search terms [S100 or S-100] as well as [S100B or S-100B] and [schizophrenia] were used to identify original studies published between 1970 and October 2015 in PubMed, Web of Science, Ovid and Scopus databases. Studies had to meet the following inclusion criteria: peer-reviewed, patients diagnosed with schizophrenia according to International Classification of Diseases (ICD-10) and/or Diagnostic and Statistical Manual of Mental Disorders (DSM-IV) standards, original studies, comparison with age-matched control subjects. Studies were checked for eligibility and selected by two persons (KS and MP). We pooled plasma and serum studies under the assumption that calculation of effect sizes normalizes absolute differences between patients and control subjects and thus, eliminates differences in methodological approaches.

### Data Synthesis

S100B levels were extracted from the articles along with information on additional covariates as examined and reported by the investigators. In case exact values were not given in the article or if data were only illustrated in plots, authors were contacted to obtain detailed information. To adjust for systematic measurement effects in the several original studies, we calculated effect sizes. Standard deviations (SD) were calculated from standard error of the mean (SEM) using the formula SD = SEM*√n if necessary. Following a conservative approach, the Comprehensive Meta-Analysis software package (versions 2 and 3, Biostat, Inc., Englewood, NJ, USA^1^) was then used to compute Hedges’ g corrected for small sample size effects under a random effects model, with effect sizes of 0.2 signifying a small, 0.5 a medium, and 0.8 and larger a strong effect (Cohen, 1988; Lakens, 2013). This software has already been used in several other meta-analytic studies (e.g., Gami et al., 2007; Hofmann and Smits, 2008; Howren et al., 2009; Leucht et al., 2009).

Besides investigating differences between patients with schizophrenia and healthy control subjects *per se*, we conducted subgroup analyses comparing drug-free with medicated patients. Additionally, we checked for possible effects of clinical covariates on effect size via meta-regression, applying the Method of Moments (DerSimonian and Laird, 1986). Potential covariates included illness duration, age at onset of the disorder, male-to-female ratio, severity of clinical symptoms as measured with the clinical symptom scales Brief Psychiatric Rating Scale (BPRS, total score), and Positive And Negative Syndrome Scale (PANSS, total score as well as positive, negative and general psychopathology subscores), as well as an index of risk of bias for cross-sectional studies published in Polyakova et al. (2015). Note that mean age was not included in this analysis, because in each of the included studies schizophrenia patients were compared with age-matched control subjects (see inclusion criteria). Body mass index (BMI), although of interest due to its potential effects on serum S100B (e.g., see Steiner et al., 2010a,b, 2014), could not yet be analyzed as a covariate due to lack of a sufficient number of studies reporting this measure.

## Results

### Identified Studies

**Figure 1:**
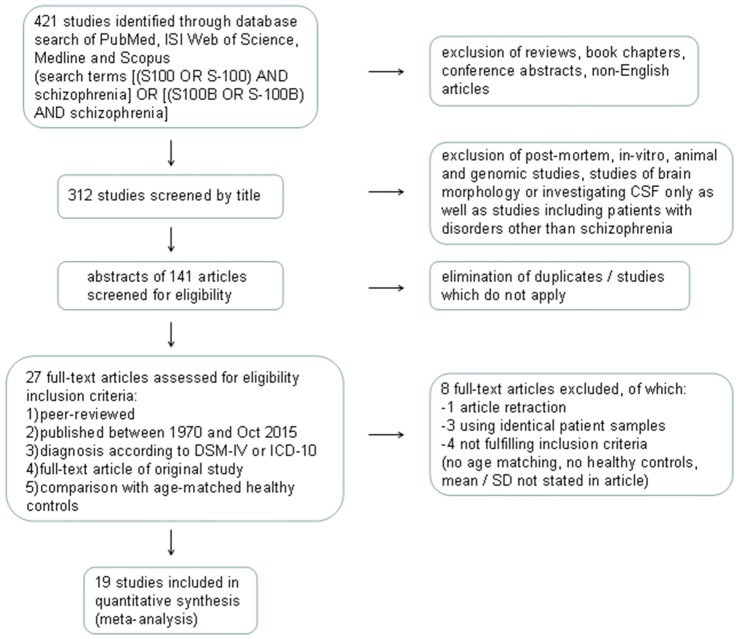
**Flow diagram illustrating the database search process according to Preferred Reporting Items for Systematic Reviews and Meta-Analyses (PRISMA).** CSF, cerebrospinal fluid; DSM-IV, Diagnostic and Statistical Manual of Mental Disorders IV; ICD-10, International Statistical Classification of Diseases and Related Health Problems 10; SD, standard deviation.

Details of the study selection process are illustrated in the PRISMA flow diagram in Figure 1. The search in PubMed yielded a total of 121 results, while Web of Science offered 149 hits, Ovid 42 and Scopus 109 results. Following exclusion of reviews, conference abstracts, oncology related papers, book chapters, errata as well as texts written in languages other than English via database settings, 312 studies remained. Further elimination by title of post-mortem, *in vitro*, animal and genetic studies, studies investigating S100B in CSF only or studies with patients other than schizophrenia resulted in 38 articles in PubMed, 39 in Web of Science, 21 in Ovid, and 43 in Scopus databases. The abstracts of these 141 articles were screened for eligibility, leading to a total of 80 articles which were then checked for duplicates, resulting in 27 full-text articles to be examined for meeting the inclusion criteria. Corresponding authors were contacted in cases where S100B levels or patient characteristics for schizophrenia-only patients were not explicitly stated in the original article. As authors did not reply, another two studies were excluded (van der Leeuw et al., 2013; Xiong et al., 2014). This resulted in an overall number of 19 original studies to be included in the quantitative meta-analysis, comprising a total of 766 patients and 607 healthy control subjects. Included studies and clinical characteristics of the study population are reported in Table 1.

**Table 1:** **Study characteristics for the 19 studies included in the final meta-analysis**.

Reference	Sample size		Patient age (years; mean ± SD)	Illness duration (years; mean ± SD)	Serum S100B (ng/l; mean ± SD)		Male-to-female ratio for patients	PANSS score				BPRS	Bias rating
	P	Co			P	Co		Total	Positive	Negative	General		
Gattaz et al. (2000)	23	23	36.0 ± 9.0	17.0 ± 7.0	440.0 ± 270.0	550.0 ± 140.0	2.3					15.8 ± 15.4	13
Ryoun Kim et al. (2007)	60	30	37.0 ± 3.5	15.0 ± 6.7	140.0 ± 91.7	78.0 ± 47.0	0.8						13
Lara et al. (2001)	20	20	31.0 ± 8.0	9.0 ± 7.0	120.0 ± 140.0	66.0 ± 67.0	1.9	107.0 ± 29.0	24.0 ± 9.0	29.0 ± 7.0	54.0		14
Ling et al. (2007)	57	60	33.5 ± 11.4	8.0 ± 9.0	119.0 ± 59.0	67.0 ± 22.0	0.9	77.8 ± 13.8		20.0 ± 6.8			14
O’Connell et al. (2013)	97	27	42.5 ± 12.2	Only clozapine duration stated	79.5 ± 39.8	67.8 ± 20.8	2.3					31.0 ± 9.0	14
Qi et al. (2009)	63	50	50.8 ± 6.8	25.4 ± 7.2	359.0 ± 116.0	123.0 ± 50.0	2.2	59.8 ± 13.1	13.2 ± 6.1	20 ± 6.2	26.5 ± 5.5		18
Rothermundt et al. (2001)	26	26	37.0 ± 12.9	10.0 ± 10.4	98.0 ± 76.0	34.0 ± 17.5	0.6	86.7 ± 17.9	25 ± 6.3	19.5 ± 8.3	42.1 ± 9.6		16
Rothermundt et al. (2004a)	21	21	32.5 ± 13.0		65.0 ± 31.0	38.0 ± 8.0	4.3	97.7 ± 25.6					13
Rothermundt et al. (2004b)	98	98	42.1 ± 11.1		73.0 ± 32.0	44.0 ± 15.0	1.3	82.5 ± 17.1	14.8 ± 5.2	27.3 ± 5.3	40.3 ± 10.6		16
Rothermundt et al. (2007)	12	12	25.3 ± 4.8	1.9 ± 1.4	85.0 ± 70.0	38.0 ± 8.0	11	81.2 ± 20.1	19.3 ± 7.4	19.6 ± 7.8	42.4 ± 11.1		18
Sarandol et al. (2007)	40	35	34.0 ± 9.9	6.7 ± 6.4	46.1 ± 43.6	23.6 ± 14.9	0.8					21.0 ± 6.1	15
Schmitt et al. (2005)	41	23	63.3 ± 7.0	35.3 ± 11.4	132.2 ± 43.0	61.0 ± 26.0	1.4					46.2 ± 14.9	16
Schroeter et al. (2003)	30	15	34.8 ± 12.4	8.9 ± 8.8	180.3 ± 129.5	112.8 ± 53.4	0.9					45.3 ± 12.6	12
Schroeter et al. (2009)	20	19	34.6 ± 12.7	8.4 ± 9.6	73.4 ± 72.1	42.1 ± 69.7	0.8					47.6 ± 11.9	15
Steiner et al. (2006)	12	17	24.0 ± 7.0	0.4 ± 0.2	90.0 ± 30.0	80.0 ± 20.0	0.7	87.0 ± 15.0	24.0 ± 6.0	21.0 ± 6.0	42.0 ± 8.0		14
Steiner et al. (2009)	26	32	34.7 ± 11.3	8.0 ± 9.0	72.0 ± 38.0	52.0 ± 18.0	1.9	84.8 ± 11.2	20.1 ± 4.9	22.1 ± 6.5	42.7 ± 5.6		16
Uzbay et al. (2013)	18	19	37.4 ± 12.6	9.8 ± 10.11	7.8 ± 10.6	6.3 ± 7.8	1.6	87.2 ± 13.3					16
Wiesmann et al. (1999)	20	20	35.7 ± 10.7	8.0 ± 5.0	165.0 ± 138.0	54.0 ± 31.0	0.7						16
Zhang et al. (2010)	82	60	50.9 ± 7.2	26.6 ± 8.7	445.3 ± 196.0	122.0 ± 76.0	2.3	58.4 ± 13.2	12.2 ± 5.9	19.9 ± 6.5	26.2 ± 5.4		18
Total study population	766	607	37.8 ± 9.2	12.4 ± 9.4	146.9 ± 126.9	88.4 ± 119.7	2.0 ± 2.4	82.3 ± 14.2	19.1 ± 5.1	22.0 ± 3.6	39.3 ± 6.3	34.5 ± 13.9	15.1 ± 1.8

*Note: BPRS, Brief Psychiatric Rating Scale; Co, control subjects; P, patients; PANSS, Positive And Negative Syndrome Scale; SD, standard deviation*.

### Main Effects

**Figure 2:**
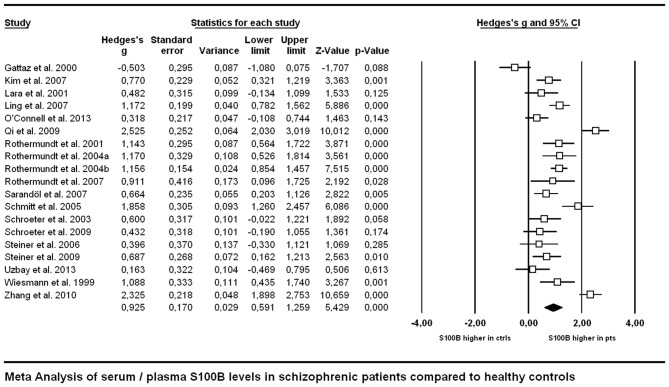
**Forest plot for the meta-analysis of serum S100B levels in schizophrenic patients compared to healthy control subjects.** Hedges’ g was used as an estimate of effect size under a random effects model. CI, confidence interval.

Across all included studies comparing patients suffering from schizophrenia with control subjects, Hedges’ g amounted to 0.925, indicating a higher level of S100B in schizophrenia patients compared to healthy controls (Figure 2).

**Figure 3:**
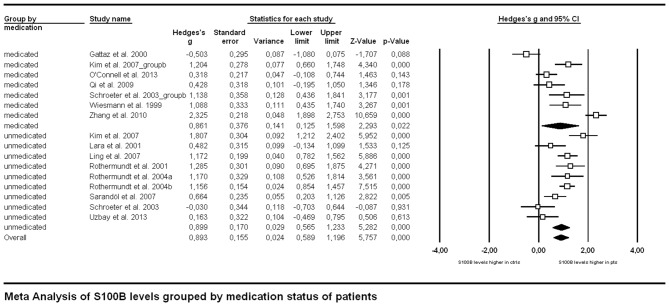
**Forest plot for the meta-analysis of serum S100B levels in medicated vs. unmedicated patients in a cross-sectional design.** Hedges’ g was used as an estimate of effect size under a random effects model. CI, confidence interval.

In order to analyze the influence of medication on serum S100B levels in schizophrenia there are generally two options. Firstly, one might compare medicated and unmedicated patients in a cross-sectional approach. Secondly, meta-analyzing longitudinal studies enables investigating treatment effects in the same cohort. Comparing studies including only medicated (*n* = 249) to those including only unmedicated patients (*n* = 244) in a subgroup meta-analysis, there was no significant difference in effect sizes between those two groups (*p* = 0.927, Figure 3). Note that studies including both medicated and unmedicated patients without analyzing them separately had to be excluded from this analysis. There were, however, high levels of heterogeneity even within the two subgroups (see Table 1) as well as a relatively small number of studies in the subgroups (seven studies for medicated, nine for unmedicated subjects, see Figure 3).

**Figure 4:**
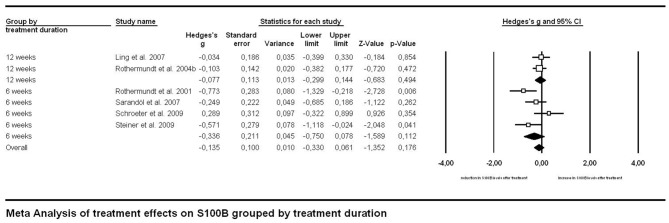
**Forest plot for the meta-analysis of serum S100B levels before vs. after neuroleptic/antipsychotic treatment in longitudinal studies.** Hedges’ g was used as an estimate of effect size under a random effects model. Effect sizes are shown for each treatment duration separately as well as the overall effect of treatment regardless of duration. CI, confidence interval.

Investigating effects of treatment with the longitudinal approach, thus meta-analyzing treatment studies within the same subjects (Figure 4), we found no significant difference in treatment effects between patient groups undergoing medication for 6 vs. for 12 weeks (*p* = 0.281). Neither did the overall treatment effect size (*g* = −0.135, S100B levels lower after treatment than before) reach significance (*p* = 0.176) in a mixed effects analysis.

### Meta-Regression

**Figure 5:**
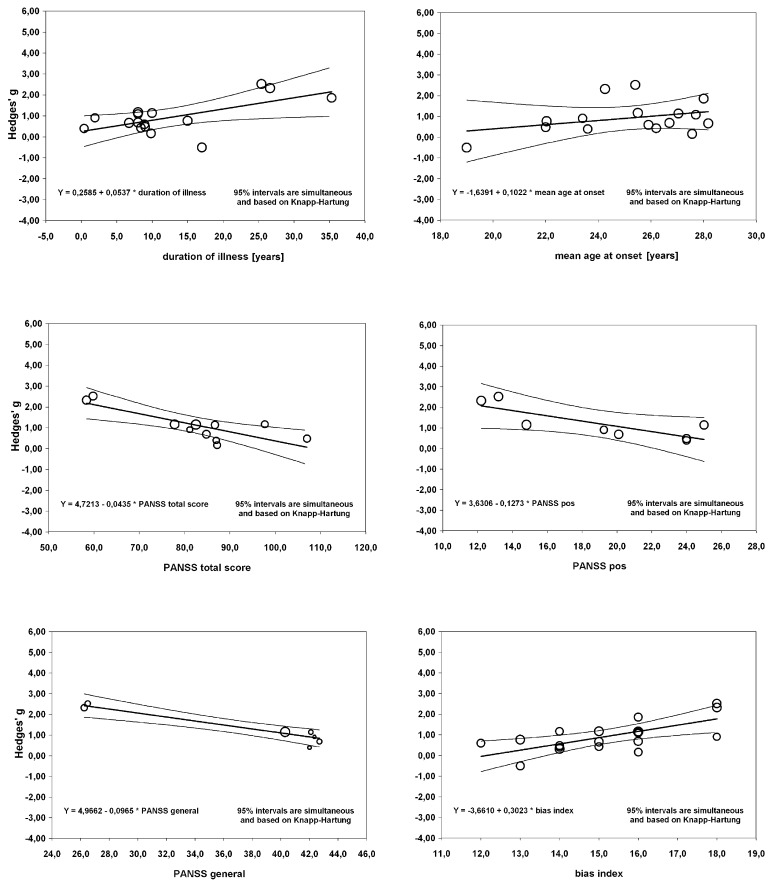
**Results of a meta-regression analysis investigating the influence of duration of illness, age at onset, PANSS total and positive score, PANSS general subscale score and bias index on effect size of serum S100B levels in schizophrenia.** PANSS, Positive And Negative Syndrome Scale.

The meta-regression of S100B serum levels with clinical parameters in schizophrenia revealed significant effects for the covariates illness duration (β_illness duration_ = 0.0537, *p* = 0.01), bias index (β_bias index_ = 0.3023, *p* = 0.001) as well as PANSS total (β_PANSS total_ = −0.0435, *p* = 0.001), positive (β_PANSS positive_ = −0.1273, *p* = 0.02) and general psychopathology (β_PANSS general_ = −0.0965, *p* < 0.001) scores, but not for any of the other regressions calculated (see Table 2, Figure 5).

**Table 2:** **Results of simple meta-regression of covariates with serum S100B effect size**.

Covariate	Number of studies	Coefficient (β)	*r*^2^	*p*		
Mean age	19	0.0573	0.52	0.0026
Illness duration	16	0.0537	0.46	0.0086
Mean age at onset	16	0.1022	0	0.2329
Male-to-female ratio	19	0.0161	0	0.8444
PANSS total score	11	−0.0435	0.82	0.0014
PANSS positive	8	−0.1273	0.55	0.0228
PANSS negative	9	−0.0856	0	0.2766
PANSS general	7	−0.0965	1	0.0008
BPRS score	6	0.0339	0.25	0.1859
Bias index	19	0.3023	0.61	0.0010

*Note: BPRS, Brief Psychiatric Rating Scale; PANSS, Positive And Negative Syndrome Scale*.

Subsequent multiple meta-regression of the PANSS subscores was then performed to obtain an impression of the influence of the individual factors while partialling out the impact of the other subscales. Results are illustrated in Table 3.

**Table 3:** **Results of multiple meta-regression analysis with serum S100B effect size**.

Covariate	Number of studies	Coefficient (β)	*r*^2^ of combined model	*p*
Illness duration	16	0.0538	0.54	0.0062
Mean age at onset		0.0997		0.1171
PANSS positive	7	−0.0038	0.98	0.9635
PANSS negative		0.0095		0.9128
PANSS general		−0.0977		0.1561
PANSS positive	8	−0.1203	0.80	0.0136
PANSS negative		−0.0797		0.1316
PANSS positive	7	−0.0127	1.00	0.6925
PANSS general		−0.0911		0.0077
Illness duration	9	0.0446	0.96	0.0685
PANSS total		−0.0284		0.0606

*Note: BPRS, Brief Psychiatric Rating Scale; PANSS, Positive And Negative Syndrome Scale*.

A multiple meta-regression analysis including the factors illness duration and age at onset revealed a significant influence of the former (β_illness duration_ = 0.0538, *p* < 0.01), while the latter failed to meet significance criteria (β_age at onset_ = 0.0997, *p* = 0.12, n.s., see Table 3). A model including the positive and the negative subscale showed a significant effect of the PANSS positive score on predicting effect sizes in the individual studies, whereas testing the influence of the negative subscale as a predictor while holding PANSS positive constant did not lead to this factor becoming significant (β_PANSS positive_ = −0.1203, *p* = 0.01, β_PANSS negative_ = −0.0797, *p* = 0.13, n.s.). A model including the positive and the general subscale revealed the factor general psychopathology to remain significant (β_PANSS general_ = −0.0911, *p* < 0.01) while the positive subscale lost its predictive value (β_PANSS positive_ = −0.0127, *p* = 0.69, n.s.). No individual factor remained significant in an analysis including all three subscales (β_PANSS positive_ = −0.0038, *p* = 0.96, n.s., β_PANSS negative_ = 0.0095, *p* = 0.91, n.s., β_PANSS general_ = −0.0977, *p* = 0.16, n.s.).

## Discussion

Our comprehensive meta-analysis, including 19 original studies with 766 patients and 607 healthy control subjects revealed elevated levels of the glial marker protein S100B in serum in schizophrenia, which is related to illness duration and to clinical symptomatology. In the following we want to discuss these findings in detail.

### Serum S100B is Increased in Schizophrenia without Influences of Medication

Regarding the comparison of patient vs. control group, the outcome of a Hedges’ g of 0.925 constitutes a rather strong effect (Figure 2), thus confirming the result of previous meta-analyses indicating higher levels of S100B in serum of patients compared to healthy control subjects (Schroeter et al., 2003; Schroeter and Steiner, 2009; Aleksovska et al., 2014). Testing the influence of risk of bias on effect size by meta-regression of a measure of bias published in Polyakova et al. (2015), we found that higher S100B effects were related to a lower likelihood of bias, as indicated by a high score in this bias index (Figure 5). Thus, studies with higher methodological quality tend to show higher S100B serum levels in patients compared to healthy controls, suggesting that this effect is not overestimated. Differences in included studies in comparison to the meta-analysis by Aleksovska et al. (2014) are related to more conservative inclusion criteria in our study, in particular the stricter age-matching of control cohorts.

Medication effects were investigated with two complementary approaches in our meta-analysis. Both the cross-sectional and the longitudinal approach revealed no significant effect of neuroleptic treatment on serum S100B levels in schizophrenia. Regarding the cross-sectional analysis one has to take into account that the lack of a between-group effect when comparing studies with only medicated to those including only unmedicated subjects could be due to a high heterogeneity both within and between studies in both groups. Unmedicated subjects encompassed never-medicated as well as patients off neuroleptics/antipsychotics for a minimum of 1 week, whereas medicated subjects widely differed in the type of neuroleptic/antipsychotic drug they were administered. Additionally, information on further psychoactive co-medication or either licit (tobacco or alcohol) or illicit drug use was not consistently supplied across studies. Similarly, the longitudinal approach also included a mixture of drug-naïve patients and such with prior neuroleptic medication but drug-free at the time of investigation. Subsequent treatment likewise consisted of different types of neuroleptics. Moreover, only six longitudinal studies were available for meta-analysis, hence lack of statistical power might be an important factor to be considered here. Accordingly, future better-controlled studies are required to disentangle the impact of medication and disease.

In sum, our meta-analyses indicate higher S100B serum levels in schizophrenia when compared to control subjects without any evidence for treatment effects to date. Our results confirm elevated S100B serum levels as an indicator of glial pathology in schizophrenia (Rothermundt et al., 2004a; Schroeter et al., 2009; Aleksovska et al., 2014), although this finding does not seem to be disease-specific, i.e., serum S100B seems to be elevated also in other psychiatric disorders such as mood disorders (Schroeter and Steiner, 2009), as shown in recent meta-analyses (Schroeter et al., 2008, 2010; Schroeter et al., 2011, 2013). In contrast to antidepressant drugs, where S100B serum levels have been discussed as indicators/biomarkers for successful treatment (Schroeter et al., 2008), as of yet there is no meta-analytic evidence for serum S100B being related to treatment success of neuroleptics/antipsychotics in schizophrenia.

### Illness Duration and Clinical Symptoms are Correlated with Serum S100B Levels in Schizophrenia

There was a strong positive correlation between effect size of S100B with duration of illness, whereas the correlation analysis with age at onset did not show significant effects. This analysis included 16 original studies, and accordingly, has to be regarded as a highly consistent and relevant finding. A multiple meta-regression analysis including illness duration and age at onset confirmed the impact of the first factor on serum S100B in schizophrenia (Table 3). Although effect sizes of serum S100B levels also correlated with mean age of schizophrenia subjects (Table 2), this effect seems to be related to illness duration (*r*_mean age/illness duration_ = 0.96, *p* < 0.001; correlation according to Pearson, two-tailed *p*) since S100B effects sizes were calculated by comparing schizophrenia subjects to age-matched control subjects, hence excluding any impact of age *per se*. Therefore, mean age was excluded from the aforementioned multiple meta-regression analysis.

Contrary to earlier studies we observed a significant correlation of serum S100B effect size with PANSS total, positive and general psychopathology, yet not with PANSS negative scores (Rothermundt et al., 2001, 2004b; Schroeter et al., 2003; Schmitt et al., 2005). A high intercorrelation of the positive subscale with the total score also suggests that the effect of the PANSS total score might be driven by high scores in the positive subscale (*r*_PANSS total/PANSS positive_ = 0.83, *p* = 0.01). An additional multiple meta-regression analysis (Table 3) including positive and negative symptoms as measured with the PANSS confirmed the impact of PANSS positive scores on serum S100B levels, whereas the same analysis with the PANSS general score included additionally revealed no significant result.

While the correlation of S100B levels with the general psychopathology subscale was even stronger than with the positive subscale, it has to be noted that only seven studies contributed to the former, whereas there was one more for the latter. Furthermore, as illustrated in Figure 5, two studies seem to predominantly drive this effect, with the other five being distributed rather evenly. Thus, at the moment it is not possible to estimate whether there truly is any relation of the general psychopathology scale to S100B levels.

Seeing that the recommended minimum number of original studies for meta-regression to gain validity has been estimated at around ten (Borenstein et al., 2009), these subscale analyses for positive, negative, and general symptoms so far remain highly speculative as they all include less than ten studies (Table 2). The analysis for the PANSS total score on the other hand seems to be more statistically reliable with eleven studies included, and not prone to the strong effects of multicollinearity observed between the individual subscores.

In conclusion, duration of illness seems to influence serum S100B levels in schizophrenia patients, with larger differences between patients and control subjects the longer the disorder has been present on average. With regards to psychopathology, the PANSS total score and with a lower evidence, the PANSS positive symptom subscale as well as the PANSS general psychopathology score are inversely correlated with effect size, i.e., the more (total, positive or general) symptoms, the smaller the difference in S100B between patients and control subjects.

One might ask whether these effects, in particular influences of illness duration and clinical scores on serum S100B effect sizes, might be interrelated. Indeed, illness duration was negatively correlated with clinical symptoms as measured with the relevant PANSS scores across studies (*r*_illness duration/PANSS total_ = −0.73, *p* = 0.03, *r*_illness duration/PANSS positive_ = −0.82, *p* = 0.03, *r*_illness duration/PANSS general_ = −0.91, *p* = 0.03, in contrast *r*_illness duration/PANSS negative_ = −0.15, *p* = 0.72). Accordingly, studies examining patients with longer duration of illness find lower psychopathology scores, which might be related to medication/treatment effects in the long-term or disease course itself. Multiple meta-regression including both duration of illness and the most reliable measure for clinical symptoms, the PANSS total score, led to both factors just narrowly missing significance, indicating some shared variance (Table 3).

The relative increase of serum S100B levels in the course of schizophrenia could be explained with dynamic glial alterations in the course of the disease. Both astrocytes and oligodendrocytes contain S100B, which may be released under conditions of reduced energy supply or cell damage (Steiner et al., 2007, 2008). Accordingly, in the context of schizophrenia, both dystrophy and swelling of astrocytes were found to progress with the duration of illness in an electron microscopic study (Kolomeets and Uranova, 2010; Bernstein et al., 2015). Moreover, oligodendrocyte-related disturbances of cerebral connectivity (Yao et al., 2015) and white matter pathology also progress over time, including a disturbed connectome organization which has been related to longitudinal changes in general functioning in schizophrenia (Whitford et al., 2007; Collin et al., 2015). Decreases in white matter volume are more pronounced than gray matter changes in the course of schizophrenia (Andreasen et al., 2011). Furthermore, in this study, white matter changes were also associated with the psychotic but not the negative symptom dimension. Interestingly, serum S100B correlates with white and not gray matter parameters in healthy subjects (Streitbürger et al., 2012).

Alternatively, these findings might be related to treatment, as patients taking neuroleptic/antipsychotic medication for longer time tend to be less symptomatic compared to recent-onset schizophrenia, thus showing lower PANSS scores but possibly also elevated S100B levels with longer duration of illness. However, so far no significant medication effect could be detected in our meta-analyses of either the cross-sectional or the few longitudinal studies, and tendencies so far seem to show a reduction of serum S100B through medication (Rothermundt et al., 2001, 2004b; Ling et al., 2007; Sarandol et al., 2007; Schroeter et al., 2009; Steiner et al., 2009, 2010c; de Souza et al., 2009; Nardin et al., 2011).

A possible explanation integrating all these findings could be a mediating effect of BMI, as long-term use of neuroleptics/antipsychotics tends to lead to weight gain, and adipocytes are among the cell types secreting S100B. This explanation is well in line with results found by Steiner et al. (2010a,b, 2014), also linking this effect to changes in insulin metabolism (Steiner et al., 2010d). Alternatively, the progression of the schizophrenic disorder itself could cause metabolic changes leading to both weight gain and altered S100B secretion (see Steiner et al., 2014). In fact, Steiner et al. (2010d), found that in their group of patients, schizophrenia was generally associated with impaired glucose tolerance, irrespective of medication status or BMI.

Unfortunately, the influence of BMI as a covariate could not yet be investigated in this meta-analysis, as there are currently only three studies (Qi et al., 2009; Steiner et al., 2009; O’Connell et al., 2013) offering that kind of information. Other correlates of adipocyte or insulin metabolism so far have only been published for the patient group of Steiner et al. (2009). Consequently, although metabolic changes associated with schizophrenia seem to offer a plausible explanation for the meta-regression findings in this analysis, further research in that direction will be needed to elucidate the exact determinants of serum S100B levels as well as the precise function of this protein in schizophrenia.

## Conclusion

In summary, our comprehensive meta-analysis including 19 original studies with a total of 766 patients and 607 healthy control subjects confirms higher values of the glial serum marker protein S100B in schizophrenia compared to control subjects. Meta-regression analyses revealed significant effects of illness duration, with higher S100B serum levels in the disorder’s course, and an impact of clinical symptomatology, in particular a negative correlation of the total score of the PANSS with serum S100B levels in schizophrenia. Accordingly, results are in line with glial pathology in schizophrenia that is modulated by illness duration and related to clinical symptomatology. Further studies are needed to investigate mechanisms and mediating factors for these findings, and replicate findings for subscales measuring clinical psychopathology by including more studies.

## Author Contributions

KS and MLS have designed the study, analyzed and interpreted the data, drafted and revised the manuscript content; KS and MP have conducted the search for relevant studies and selected studies included in the meta-analysis according to inclusion and exclusion criteria. All authors have critically reviewed the manuscript and approved its final version. All authors agree to be accountable for all aspects of the work in ensuring that questions related to the accuracy or integrity of any part of the work are appropriately investigated and resolved.

## Conflict of Interest Statement

The authors declare that the research was conducted in the absence of any commercial or financial relationships that could be construed as a potential conflict of interest.

## Footnotes

^1^https://www.meta-analysis.com/
